# Cortical Response Variation with Different Sound Pressure Levels: A Combined Event-Related Potentials and fMRI Study

**DOI:** 10.1371/journal.pone.0109216

**Published:** 2014-10-03

**Authors:** Irene Neuner, Wolfram Kawohl, Jorge Arrubla, Tracy Warbrick, Konrad Hitz, Christine Wyss, Frank Boers, N. Jon Shah

**Affiliations:** 1 Institute of Neuroscience and Medicine 4, INM 4, Forschungszentrum Jülich, Jülich, Germany; 2 Department of Psychiatry, Psychotherapy and Psychosomatics, RWTH Aachen University, Aachen, Germany; 3 JARA – BRAIN – Translational Medicine, RWTH Aachen University, Aachen, Germany; 4 Centre of Social Psychiatry, Department of Psychiatry, Psychotherapy and Psychosomatics, University Hospital of Psychiatry, Zurich, Switzerland; 5 Department of Neurology, RWTH Aachen University, Aachen, Germany; University of Salamanca- Institute for Neuroscience of Castille and Leon and Medical School, Spain

## Abstract

**Introduction:**

Simultaneous recording of electroencephalography (EEG) and functional magnetic resonance imaging (fMRI) provides high spatial and temporal resolution. In this study we combined EEG and fMRI to investigate the structures involved in the processing of different sound pressure levels (SPLs).

**Methods:**

EEG data were recorded simultaneously with fMRI from 16 healthy volunteers using MR compatible devices at 3 T. Tones with different SPLs were delivered to the volunteers and the N1/P2 amplitudes were included as covariates in the fMRI data analysis in order to compare the structures activated with high and low SPLs. Analysis of variance (ANOVA) and ROI analysis were also performed. Additionally, source localisation analysis was performed on the EEG data.

**Results:**

The integration of averaged ERP parameters into the fMRI analysis showed an extended map of areas exhibiting covariation with the BOLD signal related to the auditory stimuli. The ANOVA and ROI analyses also revealed additional brain areas other than the primary auditory cortex (PAC) which were active with the auditory stimulation at different SPLs. The source localisation analyses showed additional sources apart from the PAC which were active with the high SPLs.

**Discussion:**

The PAC and the insula play an important role in the processing of different SPLs. In the fMRI analysis, additional activation was found in the anterior cingulate cortex, opercular and orbito-frontal cortices with high SPLs. A strong response of the visual cortex was also found with the high SPLs, suggesting the presence of cross-modal effects.

## Introduction

### Electrophysiology, functional imaging and multimodal techniques

Simultaneous recording of electroencephalography (EEG) and functional magnetic resonance imaging (fMRI) has shown a number of advantages that make this multimodal technique superior to fMRI alone [1]–[7]. Recording these multiple measures of brain activity at the same time, under the same physiological and psychological conditions is advantageous for many aspects of cognitive neuroscience, in particular, pharmacological challenge studies, sleep studies, studies investigating epilepsy or evoked potential studies [3], [8], [9].

Functional MRI is based on the blood oxygenation level-dependent (BOLD) contrast [10], which represents an indirect measure of brain activity. The changes in signal intensity during the experiments reveal only the haemodynamic response of brain regions that are supposed to be involved in the response to certain stimuli. The model of the haemodynamic response posits that there is a delay between stimulus and response, which varies up to 6 seconds. Thus, fMRI is a technique with high spatial resolution, but lacks good temporal resolution. On the other hand, EEG is a direct measure of neuronal activity and provides an effective means of measuring neuronal firing. It requires the synchronous activity of a large number of neurons to generate measurable electric potentials, although it has the intrinsic problem of source localization uncertainly caused mostly by the skull and the inverse problem itself, therefore lacking of good spatial resolution.

As a consequence, simultaneous EEG-fMRI has gained attention providing improved temporal and spatial resolution. It has been suggested that the BOLD signal is governed by local field potentials [11], which are also regarded to be the basis of neuronal signalling assessed by EEG. Nevertheless, the exact mechanism of coupling between the haemodynamic response measured by BOLD/fMRI and the underlying neuronal activity is poorly understood and is still an area of discussion [12]–[17].

The fundamental assumption of any integration approach is that the signals recorded with both modalities are produced by closely interacting, or at least partly overlapping, brain structures. EEG is a selective measure of current source activity, whereas the haemodynamic fMRI signal is related to energy consumption of neural populations. Simultaneous acquisition of EEG and fMRI is recognized as a combination of complementary techniques, and gives rise to the question about the best method to achieve integration during data analysis. An approach by Ostwald et al. [6] with visual evoked potentials demonstrated the success of using the properties of the EEG signal to predict changes in the BOLD response in the statistical framework of the general linear model (GLM). This is the so-called ‘integration by prediction’, where typically some feature from the EEG (e.g. alpha power, P300 amplitude) is convolved with a canonical haemodynamic response function and used as a predictor of haemodynamic activity in a GLM. Integration-by-prediction is based on the assumption that the haemodynamic response is linearly related to local changes in neuronal activity, in particular local field potentials [11], [15], [18]. In a recent study by Juckel et al., the P300 amplitudes were used in an EEG-fMRI joint analysis for the investigation of the age effects on P300 [9]. This approach proved to be successful in finding specific P300-related BOLD responses in the functional data analysis.

An important challenge for the combination, however, is the highly contaminated signal when EEG is acquired in the MR scanner. However, a number of techniques have been proposed to suppress the gradient and pulse artefacts [19]–[21].

### Auditory evoked potentials and their clinical significance

Auditory evoked potentials (AEPs) are a subclass of event-related potentials (ERPs). ERPs are defined as brain responses which are time-locked to some event, such as a sensory stimulus. Averaged ERPs are thought to originate from synchronous activity in pyramidal cells in the activated areas. ERPs result mainly from the summation of cortical excitatory and inhibitory post-synaptic potentials triggered by the release of neurotransmitters such as GABA and glutamate into the synaptic cleft [22]. Recent neurophysiological evidence supports the notion that the features of ERPs result from activity in several cortical sources that are intrinsically connected [23]. The change in amplitude of the AEPs in response to various sound pressure levels (SPLs) is referred to as loudness dependence of auditory evoked potentials (LDAEP) [24], and is considered as a measure of serotonergic activity. [25]–[28] Moreover, there are hints of the influence of other neurotransmitters such as dopamine and nitric oxide [29], [30]. Literature suggests that a pronounced LDAEP of the N1/P2 components reflects low central serotonergic neurotransmission [26]. The inverse relationship between LDAEP and central serotonergic activity has been shown by different methods and in different psychiatric disorders [27], [31]–[35].

Different strategies have been used to determine the LDAEP. Dipole source analysis (DSA) and single electrode approaches are the most used methods [36]. DSA allows the localization of the main neuronal generators in the cortex by estimating intracerebral sources for surface scalp-recorded waveforms [37]. In LDAEP studies this can indirectly be achieved using a multichannel-EEG with source analysis techniques [26], [38]–[41]. These techniques require special expertise and have been considered to be too time-consuming [31]. Thus, many studies use only one or a few EEG channels at central sites, mostly Cz, to determine the LDAEP [42]–[44]. The Cz channel has been reported to be the best single electrode position for assessing activity of the auditory cortices if multichannel based DSA is unavailable [31].

A number of studies have shown that the change in amplitude of the AEPs is positively correlated with a growth in the cortical response [38], [45]–[47]. In a study by Mulert et al. [47], a high correlation between the loudness-dependent change of the extent of fMRI activation and the corresponding changes of the mean current source density within the same region of interest covering the primary auditory cortex (PAC) was found. Moreover, in a simultaneous EEG-fMRI approach, Mayhew et al. [48] demonstrated that the subject-mean N1 amplitude correlated with the BOLD response amplitude in the auditory cortices. Importantly, an increase in N1/P2 amplitude could imply an enlarged cortical response that might not be limited to the PAC. The aforementioned integration of EEG parameters into the analysis of fMRI data could provide additional, more detailed information on the cortical variation in the processing of different SPLs.

Nevertheless, and despite numerous investigations, the exact brain regions implicated in the processing of different SPLs remain unclear. Some evidence in the LDAEP field suggests that regions other than the PAC are involved [36]. Hagenmuller *et al.* [36] compared DSA and single-electrode estimation and found different results. In that study the authors assumed that a third source might be additionally activated, contributing to the signal obtained at the scalp electrode. Similarly, Jäncke et al., [49] found increased activation of the PAC with rising SPLs, and crucially, bifrontal activation was also found, supporting the existence of a frontal source.

### Integrating the ERP information into the LDAEP fMRI data analysis

Several studies have investigated the cortical response with different SPLs in humans (Table S1), showing that other structures apart from the PAC are involved in the processing of different SPLs. Although the loudness dependence of the cortical response has been demonstrated separately in EEG and fMRI experiments, integration of the two techniques into a unique analysis has never been accomplished in a whole brain approach. This leads to the question of which EEG parameters correlate with the extent or strength of the activation in the fMRI data. On the basis of the positive correlation between amplitude of the AEPs and extent of the BOLD signal in the primary auditory cortex demonstrated by Mulert et al. [47], we hypothesize that including the N1/P2 individual amplitudes into the fMRI analysis will show additional activated voxels that will explain the inter-subject and loudness intensity variability. Including metrics reflecting loudness dependent changes in the ERP allows us to achieve this aim by focussing our fMRI analysis on brain regions that covary with a known measure of loudness dependency. The loudness dependent modulations of the ERP components occur a few hundred milliseconds after stimulus onset, a temporal resolution that is not achieved with our fMRI data alone. The temporal resolution of the EEG data allows us to extract loudness dependent parameters and focus the fMRI analysis on the brain regions that covary with these short latency modulations of the ERP. Thus, these analyses are expected to identify the cortical structures engaged in auditory perception and processing, where the early ERP components represent this level of processing, and thus the temporal resolution of the ERPs is needed to extract information of the loudness dependency.

### Hypotheses

Based on the currently available evidence we hypothesize that cortical regions other than the PAC are implicated in the cortical response to the different SPLs. We also hypothesize that such structures play a role in the amplitude variability of the AEPs that can be measured from the scalp during an LDAEP paradigm.

Thus, here we intend to answer the following questions through simultaneous EEG-fMRI measurements:

- Does the integration of the N1/P2 amplitudes into the fMRI analysis result in an extended map of the brain responses during an LDAEP paradigm in comparison to an ‘uninformed’ fMRI analysis?
- Which regions are involved in the processing of different SPLs?
- Which differences can be observed in the brain response to low and high intensity tones?

## Methods

### Subjects and measurements

During a single session measurement, EEG was recorded simultaneously with fMRI using MR compatible devices from 16 healthy volunteers (10 males, 6 females, mean age = 31.06 years old, SD = 8.90). None of the volunteers reported any history of hearing disturbances. Written informed consent was obtained from all subjects and the study was approved by the Ethics Committee of the Medical Faculty of the Rheinisch-Westfälische Technische Hochschule Aachen (RWTH Aachen University). The study was conducted according to the Declaration of Helsinki.

EEG data were recorded in Brain Vision Recorder (Version 1.20, Brain Products, Gilching, Germany) using a 64-channel MR compatible EEG system including an MR compatible amplifier and a synchronisation box (Brain Products, Gilching, Germany). The EEG cap (BrainCap MR, EasyCap GmbH, Breitbrunn, Germany) consisted of 63 scalp electrodes distributed according to the 10-10 system and one additional electrode for recording the electrocardiogram (ECG). Data were recorded relative to a Fpz reference and a ground electrode was located at AFz (10-5 electrode system) [50]. Data were sampled at 5000 Hz, with a bandpass of 0.016 – 250 Hz. Impedances at all recording electrodes were kept below 10 kΩ.

FMRI data were recorded in a 3T Siemens Magnetom Tim-Trio MR scanner. For functional BOLD imaging, a T2*-weighted EPI sequence was used (TR = 2.2 s, TE = 30 ms, field-of-view = 200 mm, slice thickness = 3 mm and number of slices = 36). The functional time series consisted of 1670 volumes, the total duration of the fMRI measurement was 61.2 minutes. Anatomical images were acquired for every subject by means of a Magnetization-Prepared, Rapid Acquisition Gradient-Echo (MP-RAGE) sequence (TR = 2250 ms, TE = 3.03 ms, field-of-view = 256 × 256 × 176 mm^3^, matrix size = 256 × 256, flip angle = 9°, 176 sagittal slices with 1 mm slice thickness and a GRAPPA factor of 2 with 70 autocalibration signal lines).

The subjects were requested to lie down and relax during the measurement. A ‘Mr. Bean’ video was presented during the recording as a distraction, which is a common practice in the LDAEP studies. Four hundred tones with a frequency of 1 kHz, a duration of 40 ms but with different SPLs were presented using Presentation software (Neurobehavioral Systems, Inc, Albany, US). The SPLs were 70, 80, 90 and 100 dB. The timing and order of the tones were randomized using optseq2 (http://www.freesurfer.net/optseq/). Optseq is a tool for automatically scheduling events for rapid-presentation event-related (RPER) fMRI experiments. It allows for more stimuli to be presented within a given scanning interval at the cost of assuming that the overlap in the haemodynamic responses will be linear. The resulting ISI varied between 6.62 – 19.83 s.

There was a delay of 26 ms between the stimuli marker in the EEG recording and the actual presenting of the tones to the volunteers. The delay between the EEG marker and the tones was measured using an oscilloscope by establishing the time between the marker signal and the onset of the tones. This time was constant and was due to processing times in the sound card of the stimulation computer.

### EEG data analysis

The EEG data were processed using Brain Vision Analyzer (Version 2.0. Brain Products, Munich, Germany). Gradient correction was performed using the method proposed by Allen et al. [20] and included in Brain Vision Analyzer. Down-sampling to 250 Hz and a low-pass filter with a cut-off frequency of 40 Hz were applied. Further filtering was applied to the data, using an IIR (infinite impulse response) filter with a low cut-off of 0.16 Hz and a high cut-off of 20 Hz. The data were re-referenced afterwards to an average reference. For correction of the pulse artefact, the heartbeat events were first detected and marked in the ECG channel, where a pulse template was sought between 0 and 10 s. The detection method was carried out in semiautomatic mode, where non-detected heartbeat events were visually identified and marked. The artefact subtraction was carried out using the method proposed by Allen et al. [19] included as toolbox in Brain Vision Analyzer, where the time delay was automatically estimated over the whole data set. Extended Infomax independent component analysis (ICA) [51] was applied to the EEG data in order to obtain independent components, and those related to ocular and muscle artefacts were removed using the ‘Inverse ICA’ tool included in Brain Vision Analyzer. The selection of the artefactual components was performed by a trained operator, The data were later segmented around the event markers, 100 ms before the onset time and 500 ms after. Segments with residual artefacts were automatically excluded using the following amplitude parameters: amplitudes of more than −80 µV, or 80 µV, respectively, were considered as artefacts. The non-excluded segments were later averaged. Two peaks were detected and visually confirmed in semiautomatic mode at Cz channel: N1 (negative polarity and latency between 100 and 180 ms) and P2 (positive polarity and latency between 190 ms and 275 ms). For the detection of the peaks, the delay between the marker and the presentation of the stimuli was taken into account. The amplitudes and latencies at individual levels were exported for statistical analysis and construction of covariants for the fMRI analysis.

### Electrophysiological cortical mapping of the ERPs

The averaged ERPs identified in the EEG data were additionally analysed using standardized low resolution brain electromagnetic tomography (sLORETA) [52] in order to identify the localisation of the underlying source generators. sLORETA is a method to estimate the localisation of brain function at specific time windows by providing a solution to the inverse problem. Technical details of sLORETA are described elsewhere [52].

Statistical differences of the cortical activity with high (90 and 100 dB) and low (70 and 80 dB) SPLs were computed as images of voxel-by-voxel t-values. Changes in cortical activity with high and low SPLs were estimated for all subjects at the time window of 140 – 280 ms post-stimulus (N1 – P2 time window). The localisation of the differences was based on the standardized electric current density and resulted in 3-dimensional t-score images. Voxels with statistically significant differences were identified using a nonparametric permutation test [53] thresholded at the 5% probability level (p <0.05) determined by 5000 randomizations. The results were controlled for type I errors arising from multiple comparisons [53].

### FMRI data analysis

FMRI images were analysed using FMRI Expert Analysis Tool (FEAT), included in FSL (Version 5.0.4. FMRIB's Software Library, www.fmrib.ox.ac.uk/fsl/). Individual pre-processing consisted of motion correction using MCFLIRT [54], brain extraction using BET [55], spatial smoothing using a Gaussian kernel of full-width at half maximum (FWHM) of 5 mm, and high-pass temporal filtering with a period of 100 s. FMRI volumes were registered to the structural scan of the individuals and also to a standard space (MNI152) using FMRIB's Nonlinear Image Registration. Z (Gaussianised T/F) statistic images were thresholded using clusters determined by Z> 2.3 and a (corrected) cluster significance threshold of p = 0.05. The subject-level model included 4 regressors, one for every type of stimulus (tone intensity), and double gamma-HRF as convolution. The GLM model included contrasts to obtain the mean of each stimulus type: 70 dB (1 0 0 0), 80 dB (0 1 0 0), 90 dB (0 0 1 0) and 100 dB (0 0 0 1) for each individual subject. Motion parameters were included in the model in order to correct for motion artefacts.

A higher-level analysis was carried out using FLAME (FMRIB's Local Analysis of Mixed Effects) stage 1 and stage 2 [56]–[58]. The first level contrasts described above were included in the GLM model. In addition the N1/P2 amplitudes were included as covariants in order to explain activation related to inter-subject BOLD signal variation with the four SPLs as follows: mean group effect at 70 dB (1 0 0 0 0 0 0 0) and N1/P2 at 70 dB (0 1 0 0 0 0 0 0), mean group effects at 80 dB (0 0 1 0 0 0 0 0) and N1/P2 at 80 dB (0 0 0 1 0 0 0 0), mean group effects at 90 dB (0 0 0 0 1 0 0 0) and N1/P2 at 90 dB (0 0 0 0 0 1 0 0), mean group effects at 100 dB (0 0 0 0 0 0 1 0) and N1/P2 at 100 dB (0 0 0 0 0 0 0 1). Thus, two contrasts were obtained for each SPL, one containing the mean activation of the group and the other containing the covariant influence on the group. Statistic images were thresholded using clusters determined by Z> 2.0 and a corrected cluster significance threshold of p = 0.05. For the comparison of low (70 and 80 dB) and high (90 and 100 dB) intensity tones two additional contrasts were calculated: an EEG-informed analyses of low> high (1 1 1 1 −1 −1 −1 −1) and high> low (−1 −1 −1 −1 1 1 1). See Figure S1.

A repeated measures analysis of variance (ANOVA) was also performed across subjects in order to investigate brain regions involved in the variability of responses at different sound intensities. The model included 1 factor (tones) at 4 levels (70 dB, 80 dB, 90 dB and 100 dB). The contrasts included were those generated by the fMRI single-level analysis. The calculation of the ANOVA was carried out using FLAME stage 1 [56]–[58]. Statistic images were thresholded using clusters determined by Z> 2.3 and a corrected cluster significance threshold of p = 0.05.

### Region of Interest analysis

In order to perform a region-of-interest (ROI) analysis, masks of ROIs were created in FSLVIEW (FMRIB's Software Library, http://fsl.fmrib.ox.ac.uk/fsl/fslview/) using the Harvard-Oxford cortical structural atlas (Harvard Center for Morphometric Analysis, Massachusetts, US). The ROIs were those structures involved in sound perception, as well as the neighbouring areas and some frontal sources described in the literature [59]. The selected ROIs were the following: anterior cingulate cortex (ACC), medial frontal cortex (MFC), Heschl's gyri, bilateral insular cortices, bilateral orbito-frontal cortices (OFC), and frontal operculum. The number of activated voxels within ROIs was extracted from the contrasts generated by the higher-level analysis using fslstats script (FSLUTILS, http://fsl.fmrib.ox.ac.uk/fsl/fslwiki/).

## Results

### EEG data

**Figure 1:**
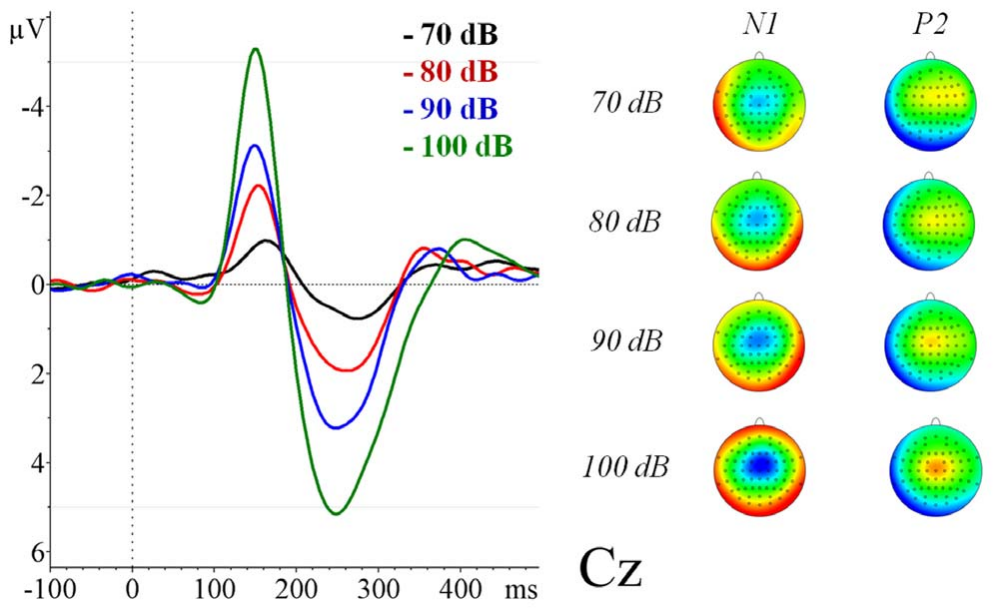
Grand average (n = 16) of the auditory evoked related potentials for the different sound pressure levels at Cz measured inside the scanner.

The EEG data were successfully corrected for gradient and pulse artefacts and the trial-average showed clear AEPs for the four SPLs (Figure 1). Descriptive statistics of ERPs at each SPL are summarized in Table 1. The mean N1/P2 amplitude of the tones at 70 dB was 2.16 µV (SD = 1.84), at 80 dB was 4.53 µV (SD = 2.72), at 90 dB was 6.86 µV (SD = 3.59) and at 100 dB was 11.26 µV (SD = 4.21). Increasing amplitudes were observed at higher SPLs, proving that the stimulation paradigm was successful in exhibiting LDAEP. See Figure S2.

**Table 1:** Descriptive statistics of N1 and P2 ERPs.

Peak and parameter	N	Mean	Std. Deviation	Min.	Max.
*Latency N1-peak 70 dB stimuli (ms)*	16	170.00	18.53	136.00	204.00
*Latency N1-peak 80 dB stimuli (ms)*	16	157.50	11.85	132.00	176.00
*Latency N1-peak 90 dB stimuli (ms)*	16	149.75	8.13	136.00	164.00
*Latency N1-peak 100 dB stimuli (ms)*	16	150.25	5.46	140.00	156.00
*Amplitude N1-peak 70 dB stimuli (ìV)*	16	−1.23	0.78	−2.44	0.57
*Amplitude N1-peak 80 dB stimuli (ìV)*	16	−2.38	1.56	−4.80	−0.01
*Amplitude N1-peak 90 dB stimuli (ìV)*	16	−3.26	1.72	−6.69	−0.96
*Amplitude N1-peak 100 dB stimuli (ìV)*	16	−5.40	2.59	−8.90	−0.90
*Latency P2-peak 70 dB stimuli (ms)*	16	253.00	28.11	192.00	288.00
*Latency P2-peak 80 dB stimuli (ms)*	16	251.00	20.26	200.00	276.00
*Latency P2-peak 90 dB stimuli (ms)*	16	250.50	20.96	220.00	292.00
*Latency P2-peak 100 dB stimuli (ms)*	16	253.75	23.60	212.00	296.00
*Amplitude P2-peak 70 dB stimuli (ìV)*	16	0.93	1.35	−0.71	4.35
*Amplitude P2-peak 80 dB stimuli (ìV)*	16	2.14	1.62	0.16	6.72
*Amplitude P2-peak 90 dB stimuli (ìV)*	16	3.61	2.32	−0.34	8.96
*Amplitude P2-peak 100 dB stimuli (ìV)*	16	5.86	2.68	1.42	11.70

### Electrophysiological cortical mapping of the ERPs

**Figure 2:**
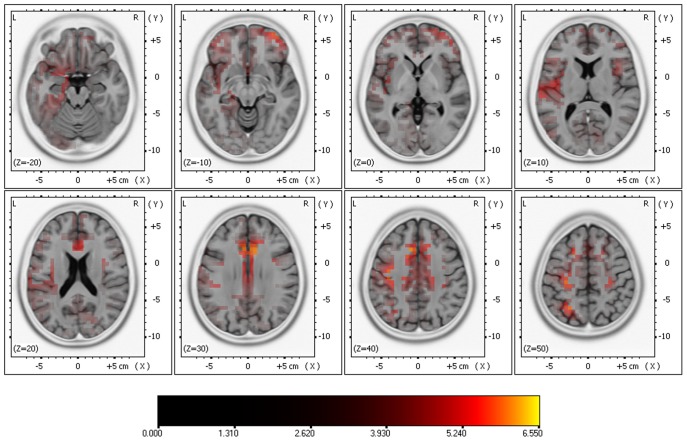
Results of comparing high vs. low SPLs in the source localization anaylsis using sLORETA. Significant differences (two-tailed, p <0.05) at 140 – 280 ms post-stimulus.

The comparison of cortical responses with low (70 and 80 dB) and high (90 and 100 dB) SPLs exhibited significant differences (two-tailed, p <0.05) at 140 – 280 ms post-stimulus. There was significantly higher activation with the high SPLs in the inferior frontal gyrus, precentral and postcentral gyri, superior temporal gyrus, superior and inferior parietal lobes, parahippocampal gyrus, ACC, uncus, insula, superior and middle frontal gyri (See Figure 2). There was no statistically significant higher activation with the low SPLs.

### FMRI data

**Figure 3:**
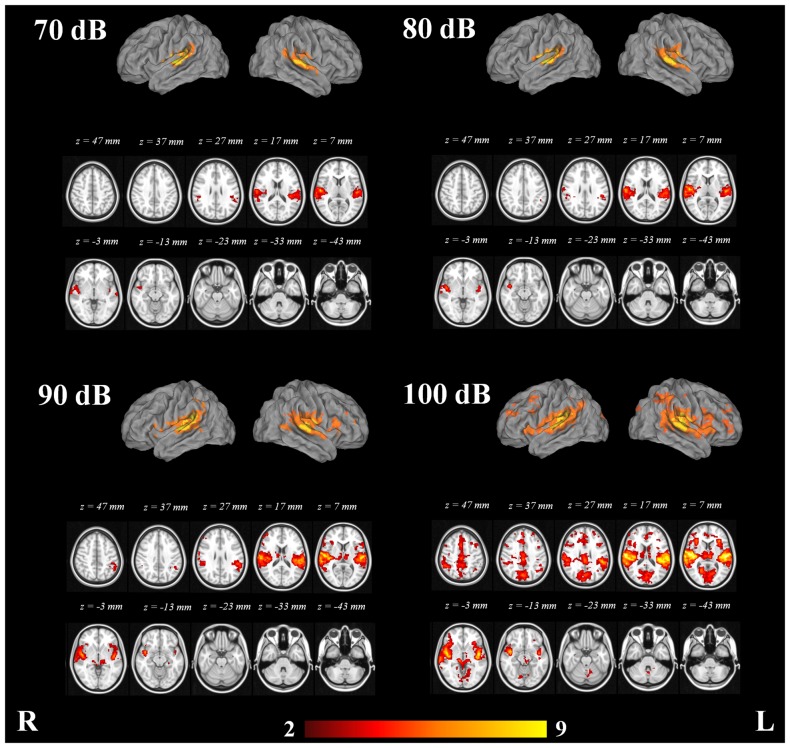
Mixed effects group analysis (n = 16) of the fMRI activation related to the 70, 80, 90 and 100 dB stimuli. Color bar represents Z-scores.

The structures which exhibited consistent activation with the four SPLs were the angular gyri, central opercular cortices, frontal operculum cortices, Heschl's gyri, insular cortices, middle temporal gyri, parietal operculum cortices, planum polare, bilateral planum temporale, postcentral gyri, precentral gyri, superior temporal gyri, temporal pole and putamen bilaterally. See Figure 3.

The fMRI data analysis showed growth in the cortical response of the PAC with increasing SPLs. In addition to the structures activated with the 70 dB stimuli, the 80 dB stimuli elicited activation in the right amygdala, right frontal orbital cortex, right pallidum, left superior parietal lobule and right thalamus. The 90 dB stimuli elicited additional activation in the caudate nucleus, right ACC, posterior cingulate cortices, frontal pole bilaterally, hippocampus, lateral occipital cortices, lingual gyri, middle frontal gyri and the parahippocampal gyri. The 100 dB stimuli elicited additional activation in the nucleus accumbens, cuneal cortices, frontal medial cortices, right inferior temporal gyrus, intracalcarine cortices, supplementary motor cortices, occipital fusiform cortices, occipital pole, paracingulate gyri, precueneus, superior frontal gyri, supracalcarine cortices, left temporal fusiform cortex and temporal occipital fusiform cortices. Clusters of peak maxima with the 70, 80, 90 and 100 dB tones in the fMRI data analysis are presented in Table 2.

**Table 2:** Clusters exhibiting activation with the 70, 80, 90 and 100 dB tones in the fMRI data analysis. Structures defined according to the Harvard-Oxford Cortical and Subcortical Structural Atlases.

	Number of voxels	Max. Z	MNI coordinates of max.			Structures of max. (Harvard-Oxford Cortical and Subcortical Structural Atlases)
			X	Y	Z	
70 dB	2885	7.2	54	−16	10	Right Planum Temporale, Right Heschl's Gyrus (includes H1 and H2), Right Central Opercular Cortex, Right Parietal Operculum Cortex, Right Superior Temporal Gyrus, posterior division
	2269	5.74	−52	−24	8	Left Parietal Operculum Cortex, Left Heschl's Gyrus (includes H1 and H2), Left Planum Temporale, Left Central Opercular Cortex
80 dB	3966	8.26	54	−16	10	Right Planum Temporale, Right Heschl's Gyrus (includes H1 and H2), Right Central Opercular Cortex, Right Parietal Operculum Cortex
	2654	6.43	−52	−20	8	Left Parietal Operculum Cortex, Left Planum Temporale, Left Heschl's Gyrus (includes H1 and H2), Left Central Opercular Cortex
90 dB	7638	9.88	54	−16	10	Right Heschl's Gyrus (includes H1 and H2), Right Central Opercular Cortex, Right Planum Temporale, Right Planum Polare, Right Parietal Operculum Cortex
	6127	7.82	−52	−20	8	Left Parietal Operculum Cortex, Left Heschl's Gyrus (includes H1 and H2), Left Central Opercular Cortex, Left Planum Temporale
100 dB	37867	12.1	56	−14	10	Right Central Opercular Cortex, Right Planum Temporale, Right Heschl's Gyrus (includes H1 and H2), Right Parietal Operculum Cortex, Right Planum Polare, Left Heschl's Gyrus (includes H1 and H2), Left Planum Temporale, Left Central Opercular Cortex, Left Planum Polare, Left Parietal Operculum Cortex

Integrating N1/P2 amplitudes into the analysis of the fMRI data resulted in voxel-wise maps showing clusters where z-scores co-varied with the N1/P2 amplitudes in an inter-subject level.

**Figure 4:**
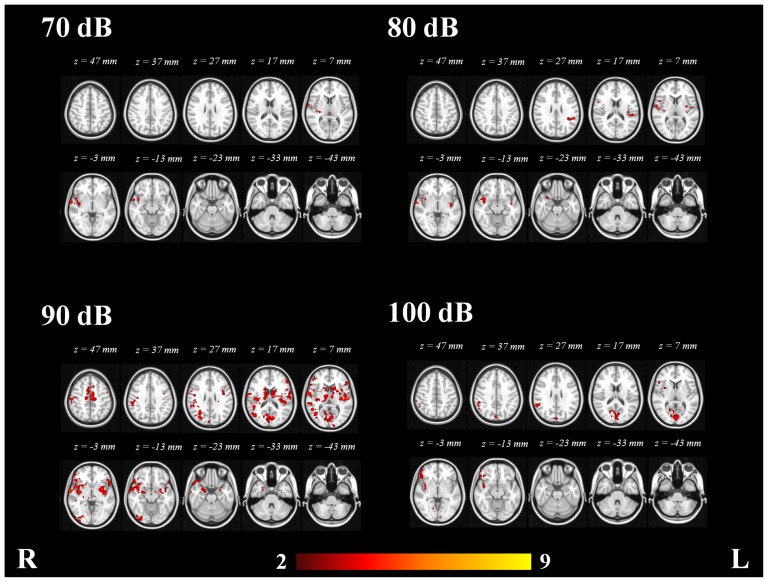
Voxel-wise statistical map of the significant clusters showing covariance with N1/P2 amplitudes at 70, 80, 90 and 100 dB. Color bar represents Z-scores.

The areas exhibiting consistent covariation of the BOLD response across subjects with the N1/P2 amplitudes with the four SPLs were the right central opercular cortices, right frontal operculum cortices, right frontal orbital cortices, right Heschl's gyrus, right inferior frontal gyrus, right insular cortex, right parietal operculum cortex, right planum polare, right planum temporale, right postecentral gyrus, right precentral gyrus, right putamen, right superior temporal gyrus and right temporal pole. See Figure 4.

In addition to the structures revealed by the group analysis of functional data, the covariate contrast of the 70 dB stimuli revealed additional activation in the right orbital cortex and right pallidum. The covariate contrast with of the 80 dB stimuli added the left caudate nucleus, pars triangularis of the right inferior frontal gyrus and right parahippocampal gyrus. The covariate contrast of the 90 dB stimuli added the nuclei accumbens, cuneal cortices, right inferior temporal gyrus, intracalcarine cortices, supplementary motor cortices, occipital fusiform gyri, occipital pole, paracingulate gyri, precuenus cortices, superior frotnal gyri, supracalcarine cortices and temporal fusiform cortices. Surprisingly when the N1/P2 amplitudes of the 100 dB tones were included into the fMRI analysis, most of the voxels which exhibited covariation were those located in the primary visual cortex (PVC), and therefore the covariate contrast did not provide additional information. Clusters with maximal covariance with N1/P2 amplitudes at 70, 80, 90 and 100 dB are presented in Table 3. A complete list of the structures which exhibited activation with the four tones is presented in Table 4.

**Table 3:** Clusters with maximal covariance with N1/P2 amplitudes at 70, 80, 90 and 100 dB in the fMRI data analysis. Structures defined according to the Harvard-Oxford Cortical and Subcortical Structural Atlases.

	Number of voxels	Max. Z	MNI coordinates of max.			Structures of max. (Harvard-Oxford Cortical and Subcortical Structural Atlases)
			X	Y	Z	
70 dB	819	4.56	62	−8	0	Right Planum Polare, Right Heschl's Gyrus (includes H1 and H2), Right Superior Temporal Gyrus, anterior division, Right Central Opercular Cortex
80 dB	863	4.32	52	−14	6	Right Planum Polare, Right Heschl's Gyrus (includes H1 and H2), Right Insular Cortex
	759	3.72	−48	−34	20	Left Heschl's Gyrus (includes H1 and H2), Left Parietal Operculum Cortex, Left Planum Temporale, Left Central Opercular Cortex
90 dB	5567	5.15	62	−6	2	Right Insular Cortex, Right Planum Polare, Right Central Opercular Cortex
	3996	4.39	−52	−18	6	Left Insular Cortex
	2220	3.92	28	−62	28	Right Intracalcarine Cortex, Right Cuneal Cortex, Right Lingual Gyrus
	1250	3.58	−6	0	54	Left Juxtapositional Lobule Cortex (formerly Supplementary Motor Cortex), Left Cingulate Gyrus, anterior division, Right Juxtapositional Lobule Cortex (formerly Supplementary Motor Cortex), Right Cingulate Gyrus, anterior division
100 dB	1156	3.56	2	−86	8	Right Supracalcarine Cortex, Right Intracalcarine Cortex, Right Lingual Gyrus, Right Cuneal Cortex, Left Supracalcarine Cortex, Left Intracalcarine Cortex
	790	3.64	40	0	−8	Right Frontal Operculum Cortex, Right Frontal Orbital Cortex, Right Inferior Frontal Gyrus, pars triangularis, Right Insular Cortex, Right Inferior Frontal Gyrus, pars opercularis
	788	3.99	52	−44	58	Right Angular Gyrus, Right Supramarginal Gyrus, posterior division

**Table 4:** Complete list of the structures (right and/or left) which exhibited activation with the four tones.

	Contrast							
Area	70 dB	70 dB N1/P2	80 dB	80 dB N1/P2	90 dB	90 dB N1/P2	100 dB	100 dB N1/P2
Accumbens	*-*	*-*	*-*	*-*	*-*	*R+L*	*L*	*-*
Amygdala	*-*	*-*	*R*	*R*	*R+L*	*R+L*	*R+L*	*-*
Angular Gyrus	*R+L*	*-*	*R+L*	*L*	*R+L*	*R*	*R+L*	*R*
Caudate	*-*	*-*	*-*	*L*	*R+L*	*R+L*	*R+L*	*-*
Central Opercular Cortex	*R+L*	*R*	*R+L*	*R+L*	*R+L*	*R+L*	*R+L*	*R*
Cingulate Gyrus, anterior division	*-*	*-*	*-*	*-*	*R*	*R+L*	*R+L*	*-*
Cingulate Gyrus, posterior division	*-*	*-*	*-*	*-*	*R+L*	*R+L*	*R+L*	*R*
Cuneal Cortex	*-*	*-*	*-*	*-*	*-*	*R+L*	*R+L*	*R+L*
Frontal Medial Cortex	*-*	*-*	*-*	*-*	*-*	*-*	*R+L*	*-*
Frontal Operculum Cortex	*R+L*	*R*	*R+L*	*R*	*R+L*	*R+L*	*R+L*	*R*
Frontal Orbital Cortex	*-*	*R*	*R*	*R*	*R+L*	*R+L*	*R+L*	*R*
Frontal Pole	*-*	*-*	*-*	*-*	*R+L*	*R+L*	*R*	*R*
Heschl's Gyrus (includes H1 and H2)	*R+L*	*R*	*R+L*	*R+L*	*R+L*	*R+L*	*R+L*	*R*
Hippocampus	*-*	*-*	*-*	*-*	*R+L*	*R+L*	*R+L*	*-*
Inferior Frontal Gyrus, pars opercularis	*R+L*	*R*	*R*	*R*	*R+L*	*R+L*	*R+L*	*R*
Inferior Frontal Gyrus, pars triangularis	*-*	*-*	*-*	*R*	*R+L*	*R+L*	*R+L*	*R*
Inferior Temporal Gyrus, anterior division	*-*	*-*	*-*	*-*	*-*	*R*	*-*	*-*
Inferior Temporal Gyrus, posterior division	*-*	*-*	*-*	*-*	*-*	*R*	*R*	*-*
Inferior Temporal Gyrus, temporooccipital part	*-*	*-*	*-*	*-*	*-*	*R*	*R*	*-*
Insular Cortex	*R+L*	*R*	*R+L*	*R+L*	*R+L*	*R+L*	*R+L*	*R*
Intracalcarine Cortex	*-*	*-*	*-*	*-*	*-*	*R+L*	*R+L*	*R+L*
Juxtapositional Lobule Cortex	*-*	*-*	*-*	*-*	*-*	*R+L*	*R+L*	*-*
Lateral Occipital Cortex, inferior division	*-*	*-*	*-*	*-*	*R+L*	*R*	*R+L*	*L*
Lateral Occipital Cortex, superior division	*-*	*-*	*-*	*-*	*R+L*	*R+L*	*R+L*	*R+L*
Lingual Gyrus	*-*	*-*	*-*	*-*	*R+L*	*R+L*	*R+L*	*R+L*
Middle Frontal Gyrus	*-*	*-*	*-*	*-*	*R+L*	*R+L*	*R+L*	*-*
Middle Temporal Gyrus, anterior division	*R+L*	*R*	*L*	*R*	*R+L*	*R*	*R+L*	*-*
Middle Temporal Gyrus, posterior division	*R+L*	*R*	*R+L*	*R*	*R+L*	*R+L*	*R+L*	*-*
Middle Temporal Gyrus, temporooccipital part	*R+L*	*-*	*R+L*	*-*	*R+L*	*R*	*R+L*	*-*
Occipital Fusiform Gyrus	*-*	*-*	*-*	*-*	*-*	*R+L*	*R+L*	*R+L*
Occipital Pole	*-*	*-*	*-*	*-*	*-*	*R+L*	*R+L*	*R+L*
Pallidum	*-*	*R*	*R*	*L*	*R+L*	*R+L*	*R+L*	*-*
Paracingulate Gyrus	*-*	*-*	*-*	*-*	*-*	*R+L*	*R+L*	*-*
Parahippocampal Gyrus, anterior division	*-*	*-*	*-*	*R*	*R*	*R+L*	*R+L*	*-*
Parahippocampal Gyrus, posterior division	*-*	*-*	*-*	*-*	*R+L*	*-*	*R+L*	*-*
Parietal Operculum Cortex	*R+L*	*R*	*R+L*	*R+L*	*R+L*	*R+L*	*R+L*	*R*
Planum Polare	*R+L*	*R*	*R+L*	*R+L*	*R+L*	*R+L*	*R+L*	*R*
Planum Temporale	*R+L*	*R*	*R+L*	*R+L*	*R+L*	*R+L*	*R+L*	*R*
Postcentral Gyrus	*R+L*	*R*	*R+L*	*R+L*	*R+L*	*R+L*	*R+L*	*R*
Precentral Gyrus	*R+L*	*R*	*R+L*	*R*	*R+L*	*R+L*	*R+L*	*R*
Precuneous Cortex	*-*	*-*	*-*	*-*	*-*	*R+L*	*R+L*	*R+L*
Putamen	*R+L*	*R*	*R+L*	*R+L*	*R+L*	*R+L*	*R+L*	*R*
Superior Frontal Gyrus	*-*	*-*	*-*	*-*	*-*	*R+L*	*R+L*	*-*
Superior Parietal Lobule	*-*	*-*	*L*	*L*	*R+L*	*R*	*R+L*	*R*
Superior Temporal Gyrus, anterior division	*R+L*	*R*	*R+L*	*R+L*	*R+L*	*R+L*	*R+L*	*-*
Superior Temporal Gyrus, posterior division	*R+L*	*R*	*R+L*	*R+L*	*R+L*	*R+L*	*R+L*	*R*
Supracalcarine Cortex	*-*	*-*	*-*	*-*	*-*	*R+L*	*R+L*	*R+L*
Supramarginal Gyrus, anterior division	*R+L*	*-*	*R+L*	*L*	*R+L*	*R+L*	*R+L*	*R*
Supramarginal Gyrus, posterior division	*R+L*	*-*	*R+L*	*L*	*R+L*	*R+L*	*R+L*	*R*
Temporal Fusiform Cortex, anterior division	*-*	*-*	*-*	*-*	*-*	*R+L*	*-*	*-*
Temporal Fusiform Cortex, posterior division	*-*	*-*	*-*	*-*	*-*	*R*	*L*	*-*
Temporal Occipital Fusiform Cortex	*-*	*-*	*-*	*-*	*-*	*-*	*R+L*	*-*
Temporal Pole	*R+L*	*R*	*R+L*	*R*	*R+L*	*R+L*	*R+L*	*R*
Thalamus	*-*	*-*	*R*	*L*	*R+L*	*R+L*	*R+L*	*-*

**Figure 5:**
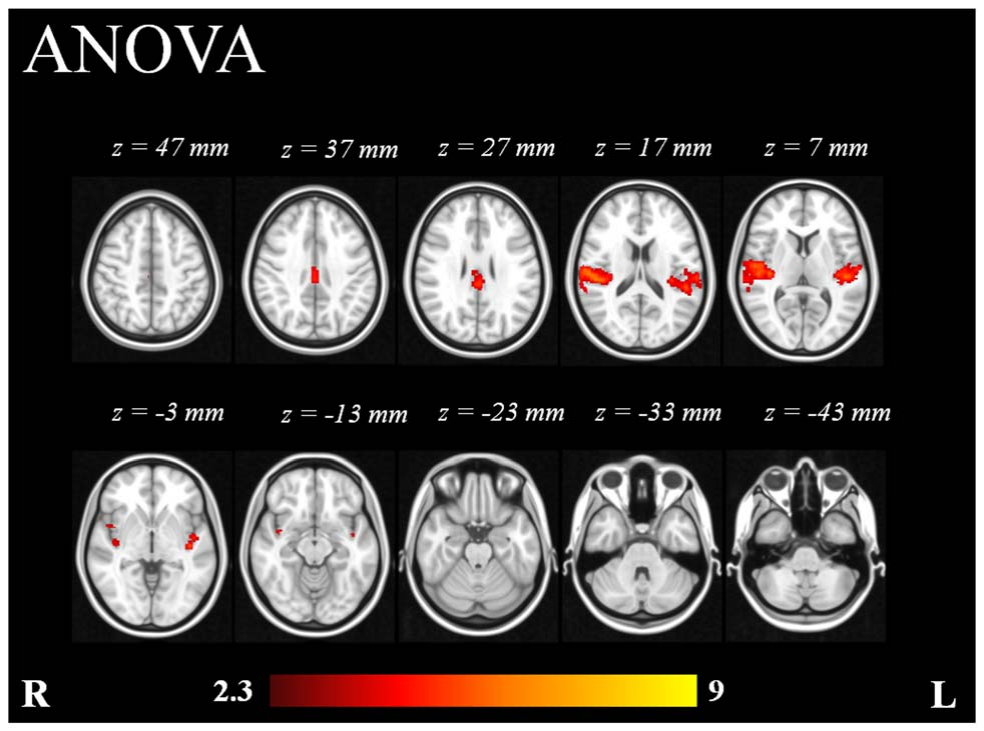
Repeated measures ANOVA analysis (n = 16) of 1 factor at 4 levels. Color bar represents Z-scores.

The ANOVA of 1 factor at 4 levels showed the brain regions whose response presented consistent variability with the different SPLs (Figure 5). The areas which exhibited consisted variation, as revealed by the ANOVA, were the Heschl's gyri (includes H1 and H2), planum temporale bilaterally, parietal operculum cortices, central opercular cortices, posterior cingulate cortex and ACC (Table 5).

**Table 5:** Results of the ANOVA analysis of 1 factor at 4 levels of 16 subjects.

Number of voxels	Max. Z	MNI coordinates of max.			Structures of max. (Harvard-Oxford Cortical and Subcortical Structural Atlases)
		X	Y	Z	
1448	4.71	50	−22	10	Heschl's Gyrus (includes H1 and H2), Planum Temporale, Parietal Operculum Cortex, Central Opercular Cortex
1306	4.74	−40	−20	0	Heschl's Gyrus (includes H1 and H2), Planum Temporale, Parietal Operculum Cortex, Central Opercular Cortex
448	3.89	0	−30	30	Cingulate Gyrus, posterior division, Cingulate Gyrus, anterior division

**Figure 6:**
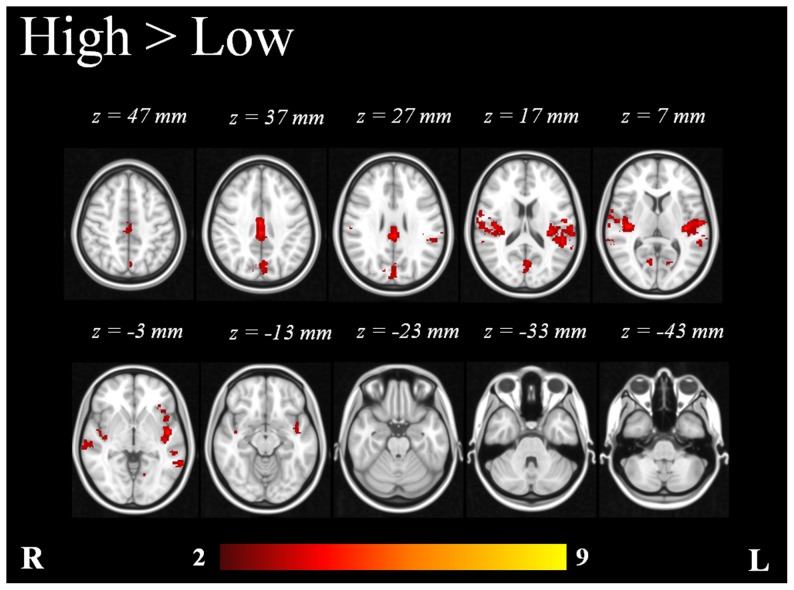
Mixed-effects statistical map of EEG-informed group analysis (n = 16) showing additional clusters in high vs. low intensity tones. Statistically significant voxels were thresholded at p <0.05. Color bar represents Z-scores.

The inclusion of the N1/P2 amplitudes resulted in an extended activation map that revealed different areas involved in the response to the tones. The voxel-wise statistical maps which resulted from the comparison of low (70 and 80 dB) and high (90 and 100 dB) intensity tones exhibited significant differences (p <0.05). The high intensity tones showed significant additional activation in right and left Heschl’s gyri, right and left insular cortices, right and left planum polare, right posterior cingulate cortex, left supramarginal gyrus, left cuneal cortex, right frontal operculum cortex, right OFC, right and left lateral occipital cortices, right and left angular gyri, left middle frontal gyrus, right postcentral gyrus and right middle temporal gyrus (Table 6 and Figure 6). The low intensity tones did not exhibit any significant additional activation in comparison to high intensity tones.

**Table 6:** Voxel-wise statistical comparison of EEG-informed High (90 and 100 dB) vs. Low (70 and 80 dB).

Number of voxels	Max. Z	MNI coordinates of max.			Structures of max. (Harvard-Oxford Cortical and Subcortical Structural Atlases)
		X	Y	Z	
2237	4.08	−40	−20	2	Left Heschl's Gyrus (includes H1 and H2), Left Planum Temporale, Left Superior Temporal Gyrus, posterior division
1252	4.44	56	−20	14	Right Heschl's Gyrus (includes H1 and H2), Right Planum Temporale, Right Central Opercular Cortex, Right Parietal Operculum Cortex
894	3.71	0	−28	38	Right Cingulate Gyrus, posterior division, Left Cingulate Gyrus, posterior division, Right Cingulate Gyrus, anterior division
738	3.02	−2	−70	18	Left Cuneal Cortex, Left Supracalcarine Cortex, Right Cuneal Cortex, Left Precuneous Cortex, Right Supracalcarine Cortex, Right Precuneous Cortex

### ROI analysis

**Figure 7:**
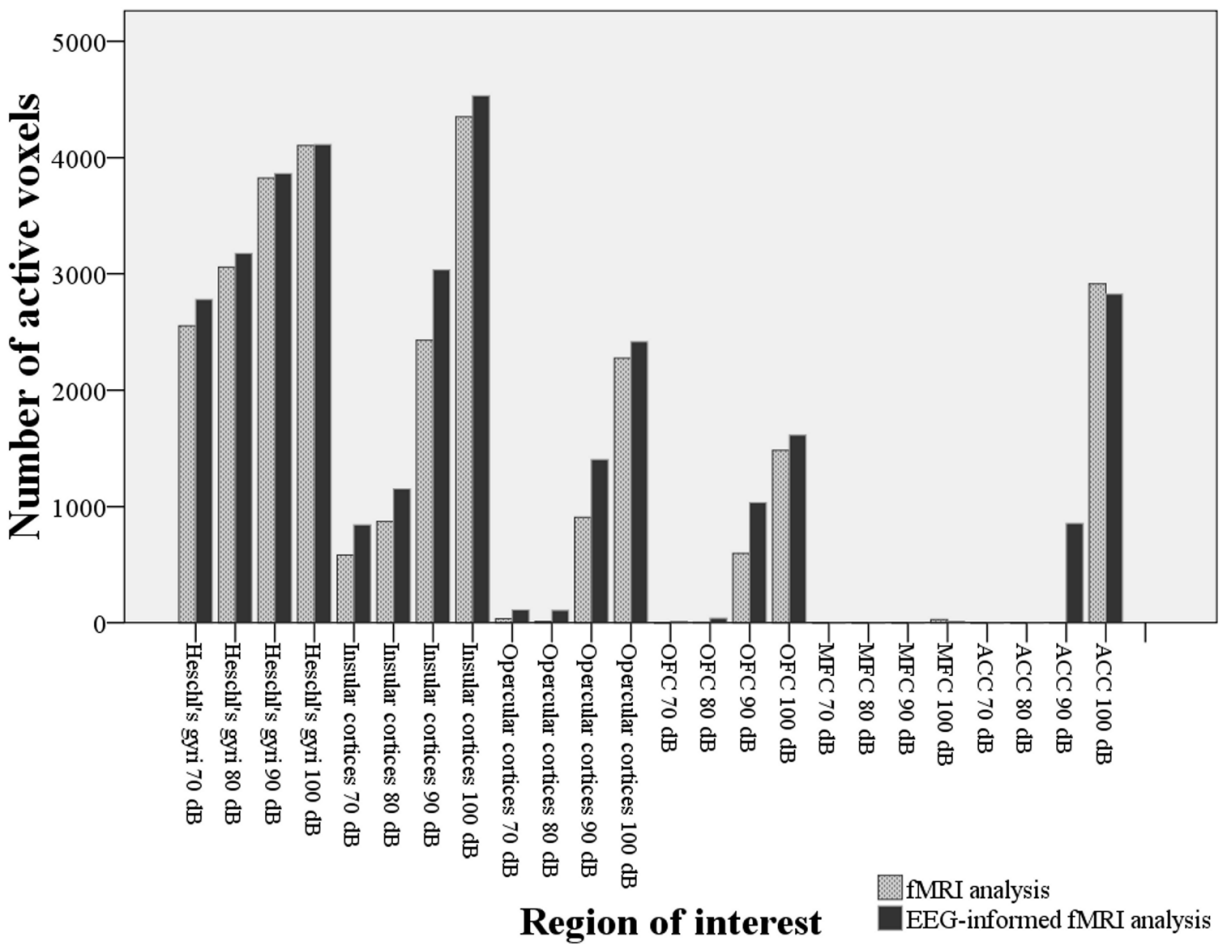
Number of activated voxels within the ROI at each sound pressure for fMRI alone analysis and EEG-informed fMRI analysis.

The ROI analysis of activated voxels within Heschl's gyri and other regions showed a greater number of voxels in the EEG-informed fMRI analysis when compared to the fMRI alone analysis (Figure 7). A paired t-test revealed this difference as statistically significant: t(23) = −3.471, p = 0.02. The analysis showed increasing activation in the Heschl's gyri and insular cortices with increasing SPLs. The EEG-informed analysis also revealed a number of activated voxels in frontal regions such as opercular cortices, OFC and ACC, particularly significant with the 90 and 100 dB stimuli. This was not the case for the MFC, which did not exhibit significant activation with any of the stimuli (Figure 7).

## Discussion

A simultaneous fMRI-EEG study was performed at 3 T in 16 healthy volunteers to investigate the cortical signal variation in the processing of different SPLs. To this end the N1/P2 components elicited during an LDAEP paradigm were extracted and included into an EEG-informed fMRI analysis. Additionally, analysis of variance (ANOVA), ROI and source localisation analyses were performed. The results are discussed in detail in the following paragraphs.

### Effectiveness of the auditory stimulation

The auditory paradigm proved to be successful for both fMRI and EEG. The presented results show a change in amplitude of the auditory evoked potentials in response to the various auditory stimulus intensities, confirming the existence of a LDAEP in our data. The paradigm was successful in terms of identifying an increase in cortical activation with increasing SPLs according to previous studies [38], [45]–[47].

### Correlation between AEP amplitudes and extent of fMRI cortical response

The main reason to combine EEG and fMRI is the synergistic effect of combining the high temporal resolution of electrophysiological measurements with the high spatial resolution of fMRI imaging [1]–[3]. The temporal resolution of the EEG data allowed us to extract loudness dependent parameters and inform the fMRI analysis with the brain regions showing covariation with such short latency modulations of the AEPs. The basic assumption in this study was that the inter-subject variability in the AEPs amplitudes covaries with the inter-subject variability of the BOLD signal [60]. The statistical maps resulting from the inclusion of the ERP-amplitudes as covariates in the fMRI data analysis, illustrate the voxels where the BOLD signal fluctuations covary with the N1/P2 amplitudes, and thus, have an influence on the inter-subject variation of the response to stimuli. Integrating the ERP parameters as predictors in the functional data allows one to reveal additional brain areas that are also important in the response to the acoustic stimuli. In line with previous work [47] this method introduces an effective way to find correlations between the cortical responses to auditory stimuli in both EEG and fMRI data. These findings also support the coupling between EEG and BOLD response during auditory stimulation described by Mayhew et al. [48].

The link between the amplitude of AEPs and BOLD signals [47] has been previously established. That is also the case for the nearly linear relationship existing between SPLs and the intensity of the BOLD signal. Nevertheless, no linear relationship between SPLs and the extent of the BOLD activation has been found, which could explain the strongest covariance of the 90 dB stimuli and the relative weak covariance of the 100 dB stimuli in the PAC [61].

### Response of cortical structures to different SPLs

The aim of this study was to identify the cortical structures that contribute to the amplitude variation of the AEPs at different SPLs. Interestingly the source localisation analysis confirmed the presence of additional activation with high SPLs. The EEG-informed fMRI analysis showed that the Heschl's gyri, as a marker of human PAC [62], were consistently activated during our paradigm with the 4 different SPLs, and that the extent of cortical response was larger at high SPLs. Other structures were also consistently activated by the stimuli, such as the angular gyri, central opercular cortices, frontal operculum cortices, insular cortices, middle temporal gyri, parietal operculum cortices, planum polare, bilateral planum temporale, postcentral gyri, precentral gyri, superior temporal gyri, temporal pole and putamen. Therefore, the inter-subject variation of the neural response to the stimuli explains the variation in amplitude that can be observed in the excitatory potentials that generate the scalp ERPs. In addition to this, the ROI analysis shows a regular pattern of increasing number of active voxels at higher sound intensities in the insular cortex.

The ANOVA pointed to the PAC, the posterior cingulate cortex and ACC as the structures that showed significant signal variation with increasing SPLs (Figure 5), suggesting that such areas play an important role in the processing of the different SPLs. The higher-level analysis confirmed the latter, since additional activation was found in the PAC and right posterior cingulate cortex with the high intensity tones (90 and 100 dB).

Interestingly, our results of the ROI and higher-level analyses show that the right and left insular cortices exhibit larger activation with high intensity tones. The insular cortex is a complex structure with several functions [63]. The association of the insular cortex with the sensory areas is well described [64]; connections have been described between the insula and the orbital cortex, frontal operculum, lateral premotor cortex and ventral granular cortex. The insula also connects with the temporal pole and the superior temporal sulcus of the temporal lobe. There is evidence that insular cortices are involved in sound detection and entry of the sound into awareness [65], [66]. In an fMRI study, Downar et al. [67] described a multimodal network for involuntary attention to events in the sensory environment that includes the insular cortex. The results of this study add an important feature to the functions of insular cortex i.e., engagement in auditory processing and also increasing response to high SPLs. Although the response of insula might not be related to sound perception, it could be related to auditory stimuli processing, which means that the increasing cortical response at higher loudness intensities is related to an intrinsic and involuntary attentional demand that is integrated in this area. Jäncke et al. [68] had already demonstrated how attention modulates activity of the PAC during auditory stimulation. Our results extend this to the insula and provide support for the function of the insula as a sensory and integrative area.

The OFC and ACC also appear in the ROI analysis as structures that exhibited activation with high intensity tones. The prefrontal cortex, especially the OFC is one of the highest order associative cortical regions of the brain. Lesions of the dorsolateral prefrontal cortex are typically associated with a number of deficits in high level cognitive processes [69]. It was also demonstrated that patients with focal lesions of the OFC had significant inhibition of startle amplitude, together with a reduced self-evaluated perception of the unpleasantness of the acoustic probe stimulus [59]. In a similar manner, the ACC has been described as part of a neural system dedicated to attention and orientation to danger, and also as an important part of the network that modulates startle responses [70].

Another interesting finding is the activation of the visual cortex particularly with the 90 and 100 dB tones. In this regard we believe that this effect was due to cross-modal effects induced by the high SPLs. There is evidence suggesting that the activity of early sensory cortex reflects perceptual experience, rather than sensory stimulation alone [36]. Supporting this assertion there is evidence of strong activation of the insular cortices in our results. In a PET study conducted by Bushara et al. [71], it was demonstrated that the insular cortex mediates temporally defined auditory-visual interaction at an early stage of cortical processing. In a similar manner, Calvert et al. [72] showed that insular cortex exhibited cross-modal interactions when the subjects were exposed to synchronous and asynchronous auditory and visual stimuli. Thus, our results show that the cross-modal effects of auditory-visual integration are stronger at high sound intensities and this integration is possibly facilitated by the increased engagement of the insula.

### Limitations

Simultaneous EEG–fMRI recordings of auditory stimulation are challenging due to the MR-acoustic environment. A number of previous studies used an interleaved experimental paradigm with quiet periods for stimulus delivery to avoid the potential confound of the MR-acoustic noise interfering with the auditory stimulus-evoked brain activity. As demonstrated by Mayhew *et al.*, [48] interleaving EEG and fMRI acquisition has important limitations, such as inefficient sampling of the neural activity as well as a decrease in the flexibility of the stimulus presentation paradigm. Moreover, the sound of the MRI scanner can induce a BOLD response in the cortical areas responsible for auditory processing, although it is largely restricted to the PAC [73].

Novitski *et al.* [74], using recorded EPI noise at 54 dB, showed no significant difference in peak-to-peak amplitude of the N1 and P2 auditory-evoked potential (AEP) peaks evoked by 57 dB pure tones and chords. Moreover, in that study it was demonstrated that the fMRI background noise does not interfere with the imaging of auditory processing related to involuntary attention.

It must be acknowledged that the EEG information used in this study was not in the temporal domain (e.g. ERP component latency) and did not exploit temporal dynamics. Nevertheless, we had a clear temporal advantage from using the ERP information in our fMRI data analysis. The ERP components of interest (N1, P2) are known to be sensitive to the loudness of auditory stimuli and our aim was to assess BOLD correlates of loudness dependency. Including metrics reflecting loudness dependent changes in the ERP allowed us to achieve this aim by focussing our fMRI analysis on brain regions that covary with a known measure of loudness dependency.

## Conclusions

The results presented here demonstrate that the extent of cortical areas involved in auditory processing rises along with rising SPLs. There was activation of the ACC, the opercular cortices and of the OFC only with high SPLs. The PAC, posterior cingulate cortex and insular cortex exhibited involvement in the processing of different SPLs. Interestingly, a strong response of the visual cortex was also found at high SPLs. We hypothesize that this is due to a cross-modal effect of the tones in the visual cortex and that it was facilitated by the integrative role of the insula.

From a methodological point of view, our study supports the suitability of including the N1/P2 amplitudes extracted from the AEPs into the analysis of fMRI data in order to enrich the results.

It could also be demonstrated that the insular cortex plays an important role in the brain response to acoustic stimulation.

## Supporting Information

Figure S1 **GLM matrix of the higher-level analysis.**
(TIF)

Figure S2 **Mean amplitude of the AEPs with the different SPLs.**
(TIF)

Table S1 **Summary of previous studies investigating the cortical response with different SPLs.**
(DOCX)
