# Supplementary figures and images for: Cortical Response Variation with Different Sound Pressure Levels: A Combined Event-Related Potentials and fMRI Study
Source: PLoS One. 2014 Oct 3;9(10):e109216. doi: 10.1371/journal.pone.0109216 (PMC4184873; doi:10.1371/journal.pone.0109216)

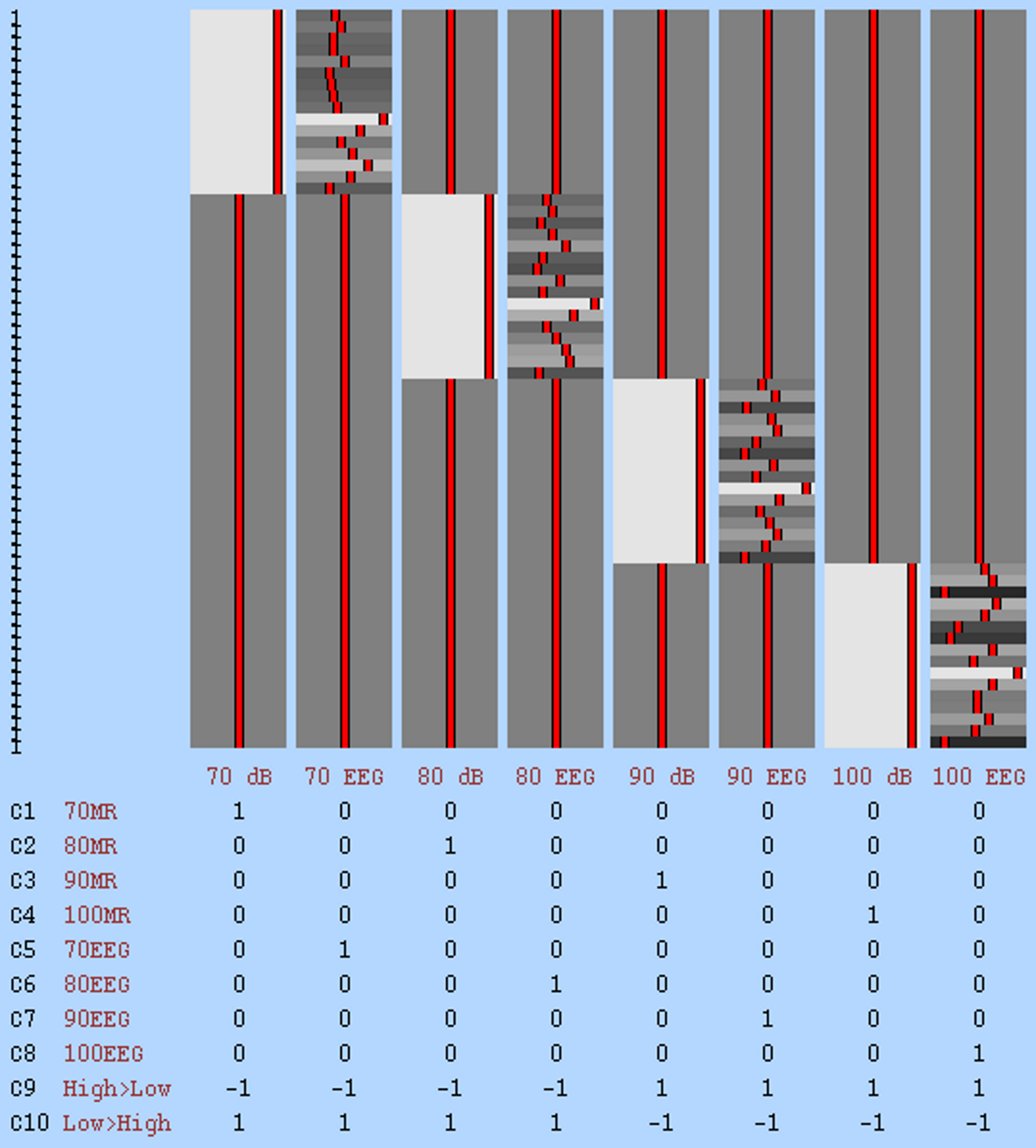

Supplement: Figure S1 — GLM matrix of the higher-level analysis. (TIF) [file pone.0109216.s001.tif]

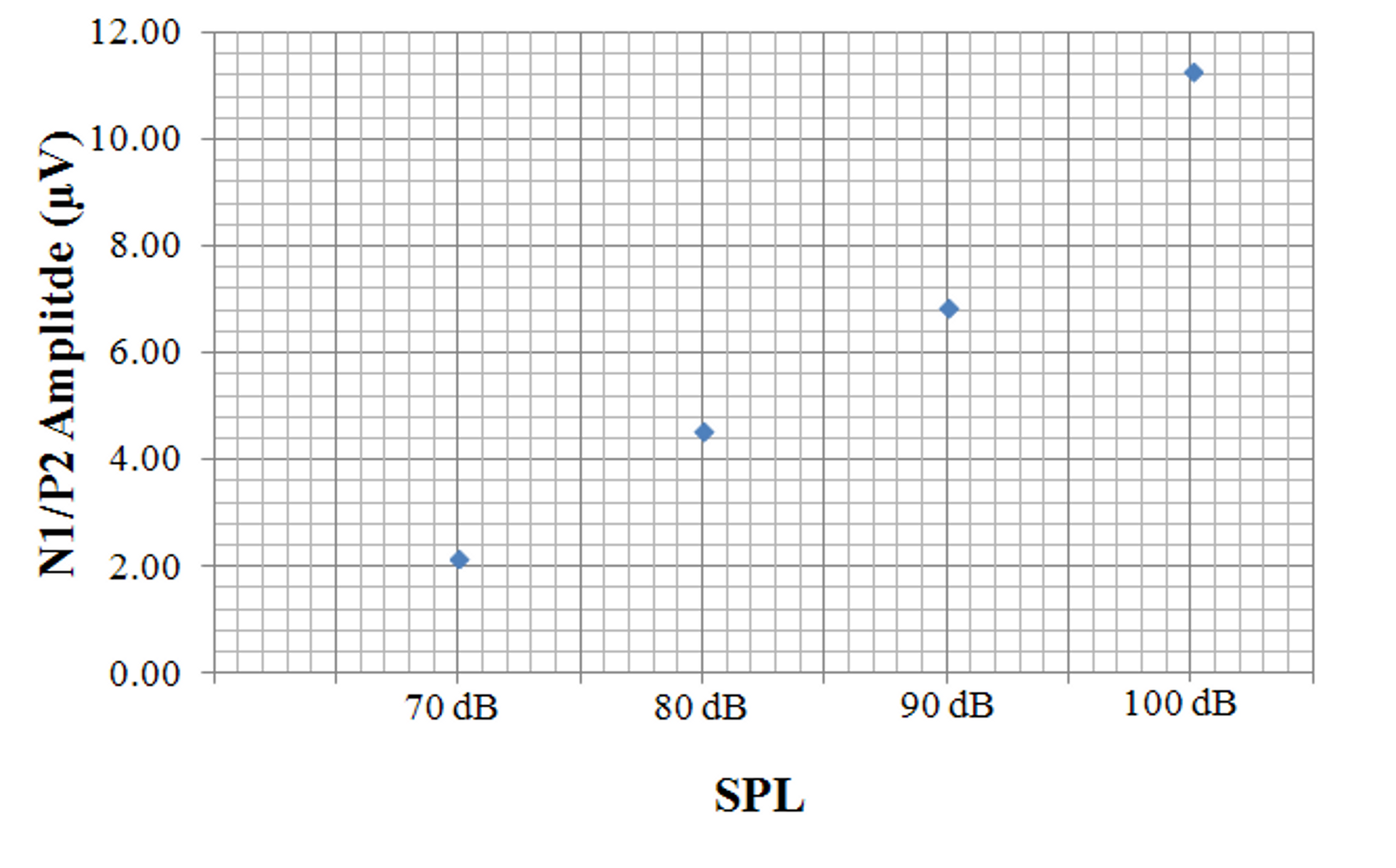

Supplement: Figure S2 — Mean amplitude of the AEPs with the different SPLs. (TIF) [file pone.0109216.s002.tif]
