# Supplementary material for: Cortical Response Variation with Different Sound Pressure Levels: A Combined Event-Related Potentials and fMRI Study
Source: PLoS One. 2014 Oct 3;9(10):e109216. doi: 10.1371/journal.pone.0109216 (PMC4184873; doi:10.1371/journal.pone.0109216)
Supplement: Table S1 — Summary of previous studies investigating the cortical response with different SPLs. (DOCX) [file pone.0109216.s003.docx]

**Table S1.**

| **Study** | **Number of volunteers** | **Technique** | **SPLs** | **Areas involved** |
| --- | --- | --- | --- | --- |
| Gallinat and Hegerl, 1994 [1] | 40 | EEG - DSA | 60, 70, 80, 90 and 100 dB | PAC |
|  |  |  |  | Lateral temporal cortex |
| Vasama et al., 1995 [2] | 6 | MEG | 40, 50, 60, and 65 dB | PAC |
| Jäncke et al., 1998 [3] | 14 | fMRI at 1.5T | 75, 85 and 95 dB | PAC |
|  |  |  |  | Inferior and middle frontal gyrus |
|  |  |  |  | Precentral gyrus |
|  |  |  |  | Middle temporal gyrus |
| Lockwood et al., 1999 [4] | 12 | [15O]H2O PET | Hearing level + 30-90 dB | PAC |
|  |  |  |  | Cochlear–olivary complex |
|  |  |  |  | Cerebellum |
|  |  |  |  | Superior temporal gyrus |
|  |  |  |  | PCC |
| Hart et al., 2002 [5] | 10 | fMRI at 3T | 42, 48, 54, 60, 66, 72, 78, 84, 90 and 96 dB | PAC |
|  |  |  |  | Precentral gyrus |
|  |  |  |  | Primary and secondary motor areas |
| Mulert et al., 2005 [6] | 17 | fMRI at 1.5T, EEG - LORETA | 60, 80, and 100 dB | PAC |
|  |  |  |  |  |
| Sigalovsky and Melcher 2006 [7] | 5 | fMRI at 3T | 30, 50 and 70 dB | PAC |
|  |  |  |  | Antero-medial area |
|  |  |  |  | Planum temporale |
|  |  |  |  | Cochlear nucleus |
|  |  |  |  | Superior olivary complex |
|  |  |  |  | Inferior colliculus |
|  |  |  |  | Medial geniculate bodies |
| Roehl and Uppenkamp, 2012 [8] | 45 | fMRI at 1.5T | Hearing level + 10, 20, 30, 40, 60, 70 and 80 dB (Max. 100 dB SPL) | PAC |
|  |  |  |  | Medial geniculate bodies |

**References**

[1] Gallinat J, Hegerl U (1994) Dipole source analysis. Linking scalp potentials to their generating neuronal structures. Pharmacopsychiatry 27: 52–53.

[2] Vasama JP, Mäkelä JP, Tissari SO, Hämäläinen MS (1995) Effects of intensity variation on human auditory evoked magnetic fields. Acta Otolaryngol 115: 616–621.

[3] Jäncke L, Shah NJ, Posse S, Grosse-Ryuken M, Müller-Gärtner HW (1998) Intensity coding of auditory stimuli: an fMRI study. Neuropsychologia 36: 875–883.

[4] Lockwood AH, Salvi RJ, Coad ML, Arnold SA, Wack DS, et al. (1999) The functional anatomy of the normal human auditory system: responses to 0.5 and 4.0 kHz tones at varied intensities. Cereb Cortex 9: 65–76.

[5] Hart HC, Palmer AR, Hall DA (2002) Heschl’s gyrus is more sensitive to tone level than non-primary auditory cortex. Hear Res 171: 177–190.

[6] Mulert C, Jäger L, Propp S, Karch S, Störmann S, et al. (2005) Sound level dependence of the primary auditory cortex: Simultaneous measurement with 61-channel EEG and fMRI. Neuroimage 28: 49–58.

[7] Sigalovsky IS, Melcher JR (2006) Effects of sound level on fMRI activation in human brainstem, thalamic and cortical centers. Hear Res 215: 67–76.

[8] Röhl M, Uppenkamp S (2012) Neural coding of sound intensity and loudness in the human auditory system. J Assoc Res Otolaryngol 13: 369–379.
